# From Sharks to Yeasts: Squalene in the Development of Vaccine Adjuvants

**DOI:** 10.3390/ph15030265

**Published:** 2022-02-22

**Authors:** Adélia Mendes, João Azevedo-Silva, João C. Fernandes

**Affiliations:** Centro de Biotecnologia e Química Fina (CBQF), Escola Superior de Biotecnologia, Universidade Católica Portuguesa, Amyris Bio Products Portugal, 4169-005 Porto, Portugal; jasilva@ucp.pt (J.A.-S.); jcfernandes@ucp.pt (J.C.F.)

**Keywords:** squalene, squalane, vaccine adjuvant, sustainability, mevalonate pathway, yeasts, industrial fermentation

## Abstract

Squalene is a natural linear triterpene that can be found in high amounts in certain fish liver oils, especially from deep-sea sharks, and to a lesser extent in a wide variety of vegeTable oils. It is currently used for numerous vaccine and drug delivery emulsions due to its stability-enhancing properties and biocompatibility. Squalene-based vaccine adjuvants, such as MF59 (Novartis), AS03 (GlaxoSmithKline Biologicals), or AF03 (Sanofi) are included in seasonal vaccines against influenza viruses and are presently being considered for inclusion in several vaccines against SARS-CoV-2 and future pandemic threats. However, harvesting sharks for this purpose raises serious ecological concerns that the exceptional demand of the pandemic has exacerbated. In this line, the use of plants to obtain phytosqualene has been seen as a more sustainable alternative, yet the lower yields and the need for huge investments in infrastructures and equipment makes this solution economically ineffective. More recently, the enormous advances in the field of synthetic biology provided innovative approaches to make squalene production more sustainable, flexible, and cheaper by using genetically modified microbes to produce pharmaceutical-grade squalene. Here, we review the biological mechanisms by which squalene-based vaccine adjuvants boost the immune response, and further compare the existing sources of squalene and their environmental impact. We propose that genetically engineered microbes are a sustainable alternative to produce squalene at industrial scale, which are likely to become the sole source of pharmaceutical-grade squalene in the foreseeable future.

## 1. Introduction

**Figure 1:**
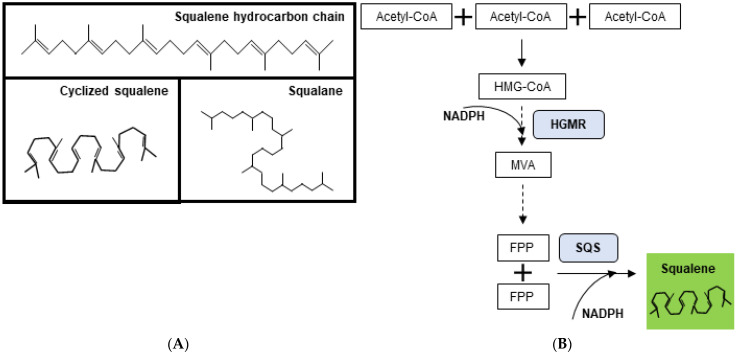
(**A**). Squalene and squalane. Structure and biosynthesis Squalene (2,6,10,15,19,23-hexamethyltetracosa-2,6,10,14,18,22-hexaene; C_30_H_50_) hydrocarbon chain (top); squalene in its cyclized form (bottom left); and fully saturated squalane (bottom right). (**B**). Simplified representation of the mevalonate (MVA) pathway in eukaryotes. Acetyl-Coa: Acetyl coenzyme A; HGM-CoA: 3-hydroxy-3-methylglutaryl-CoA; HGMR: HGM-CoA reductase; MVA: Mevalonate; FPP: Farnesyl pyrophosphate; SQS: Squalene synthase; and NADPH: Nicotinamide adenine dinucleotide phosphate. Full arrow: single step reaction; dashed arrows: simplified representation of multistep reactions.

The isoprenoid family, comprising more than 50,000 molecules, is involved in major cellular processes that include respiration, signaling, photosynthesis, and sterol and vitamin generation [1,2]. This evolutionarily conserved group of hydrocarbons derives from the 2-C-methyl-D-erythritol 4-phosphate (MEP) pathway, in prokaryotes, and the mevalonic acid (MVA) pathway, in eukaryotes [3]. Squalene (2,6,10,15,19,23-hexamethyltetracosa-2,6,10,14,18,22-hexaene; C_30_H_50_), a naturally occurring triterpene from the isoprenoid family, is an intermediate of cholesterol, phytosterol, and ergosterol synthesis in animals, plants, and fungi, respectively (Figure 1) [4]. Humans also synthesize squalene in the liver, which peaks around the second decade of life [5]. Skin and adipose tissues are the major sites for squalene storage in humans, where it accounts for up to 12% of all skin surface lipids [5,6,7].

Squalene was initially identified as a component of shark liver oil in the beginning of the twentieth century [8]. Since then, its bioactive properties have been extensively recognized. In vitro and in vivo studies unraveled squalene’s antioxidant properties by reducing stress-induced intracellular reactive oxygen species (ROS) [9]. In the skin, squalene further functions as an emollient and UV-protective agent [10,11]. Moreover, squalene decreases cytokine secretion and leads to an overall reduction in pro-inflammatory genes in immune cells, which suggests a protective role against uncontrolled inflammatory response [9,12,13]. Furthermore, as squalene is a naturally occurring oil, it has been regarded as a biocompatible drug carrier [10,14,15]. Here, squalene-gemcitabine conjugates and squalene-adenosine-α-tocopherol nanoparticles are two examples of squalene-containing formulations that, respectively, target cancer cells to enhance chemotherapy (gemcitabine) delivery and mitigate stress-induced hyperinflammation [14,15]. Still, the most widely established application of squalene in pharmaceutical formulations is in the field of vaccine adjuvants. The squalene-based oil-in-water (*o*/*w*) emulsions MF59 (Novartis), AS03 (GlaxoSmithKline, GSK), and AF03 (Sanofi) have been used as adjuvants in vaccines against influenza virus [16]. These formulations function as antigen delivery systems and potentiators of innate and adaptive immune responses (Table 1; [17,18]). In cosmetics, fully saturated squalane (C_30_H_62_), generated through squalene hydrogenation, is a valued ingredient due to its emollient, moisturizing, and antioxidant properties (Figure 1, [19,20,21]). Squalane easily penetrates human skin, hence serving as a liquid vehicle that facilitates the absorption of other bioactive molecules [22,23]. Leading cosmetic brands, such as L‘Oréal Paris and Garnier, currently include squalane in routine skin care products, such as hydrating serums and oils, and even in hair conditioner formulations.

The largest squalene natural source is the liver oil of certain fish, especially deep-sea sharks, from which it derives its name (*Squalus* spp.). Shark-derived squalene has been supplying global market demands for years since its initial discovery. However, intensive fishing had a devastating impact on marine ecosystems and endangered the populations of squalene producing shark species [19,24]. Plants also synthesize squalene (phytosqualene), albeit at lower levels than sharks. Here, oils from olive and amaranth seeds stand out as major phytosqualene providers. Nowadays, olive oil-derived squalene represents 55% of the global market, which is still insufficient to completely replace shark-derived squalene [3,22,25,26,27]. Squalene yield from plant seeds and oils is minimal (Table 2), and industrial production of phytosqualene requires expensive oil refining methods [28]. This created a market demand for alternative sources to produce squalene in a more sustainable and economically viable manner. Several microorganisms, such as bacteria, algae, and yeasts, synthesize and store squalene. When compared to sharks and plants, these microorganisms produce minimal quantities of squalene (reviewed in [3]). However, their easy maintenance, rapid life cycle and the possibility to genetically engineer and optimize their metabolism, make them appealing sources of metabolites with commercial value, such as squalene [7,21,29,30,31]. Here, the yeast *Saccharomyces cerevisiae* (*S. cerevisiae*) emerges as the standard model organism for the optimization of the isoprenoid synthetic pathway, via genetic engineering and metabolic manipulation [29,31,32].

In 2025, the squalene/squalane market is projected to value 184 million dollars (USD), with major demands expected from cosmetics and pharmaceutical industries [24]. The current pandemic context adds to squalene global needs, given the urgency to produce sufficient anti-SARS-CoV-2 vaccines to immunize the world population. This raises concerns about the sustainability of the massive production of pandemic vaccines, as shark-derived squalene has been traditionally used as an adjuvant [33]. In recent years, genetic and metabolic manipulation of microorganisms significantly improved the production of commercially appealing biomolecules, including squalene [3,21,28]. Such advances represent major breakthroughs in the supply chain of highly demanded biomolecules, potentially meeting global needs in a sustainable manner in the foreseeable future.

## 2. Squalene-Based Formulations as Vaccine Adjuvants

Vaccines are a major achievement of modern science, having saved millions of lives from infectious diseases. Vaccines are composed by pathogen components, such as protein fragments, or by live attenuated or inactivated viruses, as in influenza seasonal vaccines [16]. More recently, pathogen mRNA fragments have been used as antigens, as in the case of some anti-SARS-CoV2 vaccines recently developed [34]. Antigens are expensive to produce due to limited feedstock and to strict good manufacturing practices (GMPs) required to market a licensed antigen [35]. Additionally, the use of safer and easier to produce peptide-based subunits or nucleic acid antigens produce a relatively weak immune response and thus require the use of immunostimulants for optimal efficacy. To overcome such limitations, vaccines usually include adjuvants that enhance immunogenicity, thus ensuring an efficient immune response, using minimal quantities of antigen [18,36]. Roughly, adjuvants fall into one of three categories: (1) delivery systems that concentrate and target the antigen to antigen presenting cells (APCs); (2) immunomodulatory molecules that activate innate immunity, and (3) molecules and/or formulations combine both functions [16].

Immunocompromised populations, such as patients undergoing haemodialysis or chemotherapy, benefit the most from the inclusion of adjuvants in vaccines, as these boost their inherently weak immune response. HIV-positive patients and people in the extremes of age—the very old and the very young—have also shown significantly faster and higher antibody titers to adjuvanted vaccines than to non-adjuvanted ones [37,38]. Potassium aluminium sulphate salt (KAl(SO_4_)_2_·12H_2_O; Alum) was the first vaccine adjuvant, developed in the 1920s, and remained the sole formulation used for this purpose for more than 70 years. Nowadays, the list of licenced adjuvants continues to grow to include new molecules and formulations, such as the glycosylated triterpenes saponins, bacteria derived adjuvants, which boost innate immunity (such as the Toll-like receptor, TLRs, agonists monophosphoryl lipid A, MPLA, or CpG oligodeoxynucleotides), mineral salts, and oil emulsions such as squalene-based *o*/*w* emulsions [18,39,40].

Squalene is a naturally occurring oil in human tissues, thus it can be perceived as a secure choice to include in pharmaceutical formulations. MF59 (Novartis), AS03 (GSK), and AF03 (Sanofi) are *o*/*w* emulsions that contain squalene droplets, stabilized by non-ionic surfactants [16,18]. All three are approved for use in both seasonal and pandemic influenza vaccines (Table 1). Studies in mice and non-human primates showed that, besides their role as antigen carriers, squalene-based adjuvants enhance both innate and adaptive immune responses [17,41,42]. The biological mechanisms by which squalene-based vaccine adjuvants boost innate and adaptive immune responses have been mostly studied for MF59, the first developed squalene adjuvant. Given the similar composition of all squalene-based adjuvants, it has been generally accepted that, to some extent, both AS03 and AF03 follow an identical action mechanism to MF59 [43].

### 2.1. MF59 (Novartis)

MF59 (squalene, polysorbate 80, and sorbitan tioleate) functions as an antigen delivery vehicle and immunomodulator [17,44,45]. Despite no evidence of depot effect, MF59 muscular injection promotes local increase in chemokines and cytokines, which in turn leads to rapid immune cell recruitment [17]. The chemokine pattern induced by MF59 is distinct from other non-squalene-based adjuvants, with specific enrichment of the monocyte chemoattractant CCL2 and the neutrophil chemoattractants CCL3 and CXCL8 [45]. Despite its role as enhancer of innate immunity, MF59-induced antibody responses are activated independently of TLR and inflammasomes, via myeloid differentiation primary response 88 (MyD88; [46,47]). Innate immune responses induced by MF59 include the activation of antiviral gamma interferon (IFN-γ) signaling-related genes, with the consequent activation of monocyte and dendritic cell responses [47]. Adaptive immunity is also stimulated by MF59. In this context, studies show that the release of extracellular ATP, induced by MF59 at the site of injection, is essential for the activation of CD4^+^ T-cells [48]. Remarkably, MF59 promotes IgG isotype switch even in the absence of CD4^+^ T-cells and can induce CD8^+^ and long-lived antibody-secreting cells [44]. Compared to Alum, MF59 induces higher CD11^+^ cell recruitment, and higher neutrophil load to draining lymph nodes (dLNs; [17]). Moreover; microarray data analyses showed that MF59 induces significantly more changes in gene expression than alum and CpG adjuvants, supporting the observations that MF59 is more efficient than the latter in boosting immune response [42].

### 2.2. AS03 (GSK)

The AS03 adjuvant has a similar composition as MF59, plus α-tocopherol (Vitamin E), an enhancer of cytokine secretion and positive modulator of innate immunity [49]. AS03 is used in pandemic influenza vaccines (Table 1) and is currently under investigation for several recombinant spike protein COVID-19 vaccines [49]. Although the molecular mechanisms of AS03-induced immunity are yet to be fully disclosed, it is known to have a dose sparing effect, attributed to α-tocopherol. In the case of influenza vaccines, AS03-adjuvanted vaccines require lower antigen load than other squalene-based adjuvanted counterparts [50]. AS03 has been shown to co-localize with antigens and to induce transient innate immune responses. Locally, α-tocopherol stimulates the production of monocyte and neutrophil chemoattractants, such as CCL2 and CCL3, respectively [51]. In mice, AS03 promotes the expression of IL-6 and granulocyte colony-stimulating factor (G-CSF), via induction of the endoplasmic reticulum (ER)-stress related pathway [52]. This, in turn, leads to increased cell recruitment and antibody titers in dLNs [22]. Like MF59, AS03 further stimulates adaptive immunity. AS03-adjuvanted vaccines induced higher activation of CD4^+^ T-cell response when compared to non-adjuvanted counterparts. AS03-mediated CD4^+^ T-cell activation is accompanied by higher induction IFNγ signaling and enhanced antigen presentation in dendritic cells [53,54,55]. Strikingly, interferon upregulation by AS03 correlated with increased antibody titers 56 days after inoculation [54].

### 2.3. AF03 (Sanofi)

The AF03 adjuvant, developed by Sanofi Pasteur, is an *o*/*w* emulsion containing squalene, polyoxyethylene cetostearyl ether, mannitol, and sorbitan oleate (Table 1; [56]). The AF03 adjuvant was included in the Humenza pandemic vaccine against influenza which has, however, never been marketed [57]. The molecular mechanisms underlying AF03 immune stimulation are still largely unknown, likely due to the lack of studies on AF03-adjuvanted vaccines. To date, it has been reported AF03 potentiates IL-5 and IFN-γ immune response in mice, and that there is an increase in influenza virus neutralizing antibodies in animals inoculated with distinct AF03-adjuvanted vaccines [58,59,60]. More recently, it was reported that AS03- and AF03-adjuvanted anti-SARS-CoV2 vaccines induced higher antibody titers when compared to non-adjuvanted counterparts [61].

Today, the list of vaccine adjuvants has evolved to include a wide range of delivery vehicles, immuno-modulators, and even small molecules that target key mediators of immune response, such as TLR agonists [49]. The recent COVID-19 outbreak uncovered an inconvenient reality: traditional feedstock sources, such as shark liver oil and plant oils, are not sufficient to provide vaccines to the entire world population in a sustainable manner. The need to supply COVID-19 vaccines worldwide is expected to greatly enhance the demand for squalene, putting ecosystems at risk and increasing its cost [19,24].

**Table 1:** Squalene-based adjuvants composition and possible mechanisms of action.

Adjuvant	Vaccines	Trade Name	Hemagglutinin (HA) Dose	Possible Mechanism of Action	References
MF59 (Novartis) Squalene Polysorbate 80 Sorbitan trioleate	A/H1N1 influenza	Focetria^®^ Celtura^®^	7.5 μg/0.5 mL 3.75 μg/0.5 mL	Transient increase in local cytokines chemokines Activation of innate immunity independent of TLRs ^b^ CD4^+^ T-cell activation (dependent of extracellular ATP) Cell recruitment (neutrophils, eosinophils, and monocytes) Increase antibody titers in neutrophils in dLNs ^c^ IgG Isotype switch	[41,44,45,48]
	Seasonal influenza	FLUAD^®^ FLUAD Pediatric™	15 μg/0.5 mL ^a^ 7.5 μg/0.5 mL ^a^		
AS03 (GSK) Squalene Polysorbate 80 α-tocopherol	A/H1N1 influenza	Pandemrix^®^ Arepandix^®^	3.75 μg/0.5 mL	Spatio-temporal colocalization with antigen Direct activation of TLRs Transient increase in local cytokines chemokines Increase antibody titers in monocytes in dLNs	[51,62]
AF03 (Sanofi) Squalene Polyoxyethylene cetostearyl ether Mannitol Sorbitan oleate	A/H1N1 influenza	Humenza ^d^	Not determined	Cell recruitment to the injection site Immune response mediated by IFN-γ and IL-5	[60]

^a^ FLUAD^®^ is a trivalent inactivated influenza vaccine. HA numbers refer to the dose of each influenza strain surface antigen; ^b^ TLRs–Toll-like receptors; ^c^ dLNs–draining lymph nodes; and ^d^ Currently not in use.

## 3. Squalene Sources

Traditionally, squalene has been extracted from animal and plant sources [40]. Increasing demand, namely from cosmetic (squalane) and pharmaceutical industries, has endangered species and ecosystems [18,25]. The lack of sustainability of the squalene market fueled the search for alternatives. Genetic engineering of microorganisms, as well as the optimization of culture conditions at the industrial level, allowed several biotechnology-based companies to increase squalene feedstock in an eco-friendly and economically viable manner [25,27].

### 3.1. Squalene from Shark Liver Oil

Sharks store most of their lipid content in the liver, where the unsaponifiable matter represents around 50–80% of this organ’s weight. Most of this matter is made up of squalene [28]. Extracting squalene from shark liver oil implicates lower financial costs and it is a fairly simple process: a single distillation under vacuum at 200–300 °C is sufficient to recover more than 98% of pure squalene from shark liver oil [22]. Nevertheless, around 3000 animals are required to produce 1 ton of squalene [20]. This, together with the long reproductive cycle and slow growth rate of sharks led to a massive depletion of wild sharks, endangering some species of extinction [3,22]. Another limitation to the use of shark-derived squalene is the presence of contaminants. These include persistent organic pollutants (POPs) such as polychlorinated biphenyl (PCB), organochlorine pesticides, polycyclic aromatic hydrocarbons, dioxins, and heavy metals, all of which are bioconcentrated in the liver [3,21,28]. Moreover, the possible presence of pathogens in the liver that might be transmitted to, and infect, humans raises additional concerns about the use of shark-derived squalene [63]. Therefore, squalene purification prior to its inclusion in consumer products is essential, hence increasing its final cost. There is an increasing pressure on industry to move from shark-derived squalene to more sustainable versions, such as phytosqualene and squalene from genetically engineered organisms, such as yeast and bacteria [3,21,28]. In recent decades, several campaigns against harvesting sharks for squalene extraction have been conducted by diverse environmentally friendly organizations. In 2013, a campaign from the Environmental Justice Foundation exposed the illegal sales of shark-derived squalane, being passed off as, or blended with, olive-oil derived squalane [64].

### 3.2. Squalene from Plants

Phytosqualene, i.e., squalene obtained from plants, presents several benefits over shark-derived squalene: enhanced stability, non-greasy texture, lack of color and odor, and lighter consistency [11]. Moreover, the use of phytosqualene eliminates the risk of transmission of potentially harmful pathogens from sharks to humans. Consequently, cosmetic, and pharmaceutical industries are turning to plant-based squalene to include in skin and health-care topical formulations [20]. This may be further converted to its saturated version, squalane, for enhanced stability, spreadability onto the skin, and extended shelf-life when compared to squalene [65].

Unrefined plant oils contain minor squalene amounts. Olive oil, for example, contains up to 0.5% of squalene. However, its deodorizer distillate (DD), a by-product of the oil refining process, is enriched in bioactive molecules, such as squalene, tocopherols, phytosterols, and fatty acids [66]. Around 80% of the olive oil DD (OODD) is made up of squalene, making it a more valuable source of squalene than crude olive oil [3,28]. Among plants, the highest squalene concentration is found in the oil of *Amaranthus* species (Table 1; [28,67]). However, as the total lipid content in amaranth seeds oil is inferior to that of olive oil, the latter is still used as the main source of phytosqualene for commercial purposes [20,25,68]. Currently, research efforts are being directed towards the optimization of the extraction of high-pure squalene from amaranth seeds oil, making it a potential source for industry [68,69].

Traditional oil refining methods using organic solvents such as petroleum ether and hexane have been progressively abandoned in favor of more eco-friendly techniques [25,70]. In this context, supercritical fluid extraction using carbon dioxide (ScCO_2_) emerged as an efficient method for squalene extraction from plant oils [69,71]. The major advantage of ScCO_2_ is the ability to isolate squalene at low temperatures, thus reducing the risk of chemical modification of squalene or other oil components. Moreover, the ScCO_2_ method leaves no traces of organic solvents in the final product and results in higher squalene yield when compared to organic solvent extraction [71]. Nevertheless, using ScCO_2_ for lipid isolation implicates long processing periods and the management of complex operating parameters, such as temperature and pressure, which results in high financial costs for industries [25,28]. Consequently, some industries use a mixture of animal and plant-derived squalene, which are sometimes fraudulently labelled as vegeTable-origin squalene. Strict surveillance measures are currently enforced by the European Union to limit the fishing of endangered deep-sea animals and to precisely ascertain the origin of squalene included in distinct final consumer products [72,73].

The use of phytosqualene is not only limited by extensive and laborious oil refining processes. The seasonal nature of plant sources and geographic variations in temperature, soil composition, humidity, and pest control are major drawbacks to the mass-scale production of phytosqualene. It has been estimated that a hectare of land planted with olive trees would yield up to 50 kg of squalene [22]. Hence, there is an urgent need to identify economically viable methods to obtain squalene [20,66,74]. New squalene sources, as well as extraction methods should ensure more than 99% pure squalene (current purity level of squalene derived from shark liver oil), must be scalable for global mass production and, of upmost importance, must be eco-friendly and safeguard the sustainability of the entire extraction, purification, and production processes.

### 3.3. Microorganisms as Sustainable Sources of Squalene

Microorganisms, from yeasts to archaea, are natural sources of squalene (Table 1). Their high growth rate, together with the possibility to enhance squalene yield using genetic engineering and growing environment optimization, instigates their use as industrial sources of bioactive molecules. Squalene production by different microorganisms has been documented (Table 2). Among them, marine protists Thraustochytrids are reported as major lipid and fatty acid producers [3,75]. In particular, *Aurantiochytium* sp. can produce up to 317 mg/g dry cell weight (DCW) of squalene, the highest yield by any wild type (wt) microorganism (Table 2; [76]). Other microorganisms, such as the marine bacteria *Rubritalea squalenifaciens* sp. nov. (15 mg/g DCW), the archaea *Halobacterium cutirubrum* (1 mg/g DCW), and the oleaginous yeast strain *Pseudozyme* sp. (70.32 mg/g DCW), can also produce and store squalene [77,78,79]. The list of microorganisms capable of squalene production is increasingly widespread and diverse [3]. However, the tools to manipulate and genetically engineer much of these strains are limited, thus hampering their use in industrial context. In contrast, *Saccharomyces cerevisiae* (*S. cerevisiae*) and *Escherichia coli* (*E. coli*), are preferred experimental models for genetic manipulation, molecular and synthetic biology. Genome editing and metabolic optimization studies have generated strains that are increasingly efficient in producing commercially valuable bioactive molecules, including terpenoids such as squalene [29,30,31,32,80,81].

### 3.4. Genetic Manipulation and Metabolic Rewiring to Increase Squalene Production

*S. cerevisiae* uses 4.76 mol of glucose to produce 1 mol of β-farnesene, a direct squalene precursor [21]. In 2016, Meadows and colleagues used this yeast strain to modify the isoprenoid pathway so that it yielded 25% more β-farnesene than the native counterpart, using only 3.5 mol glucose. Replacement of NADPH-dependent HGMR by a NADH-dependent counterpart, together with the modification of key enzymes of acetyl-CoA and glucose metabolism, was central to improve pathway efficiency (Figure 1B). As a result, yeast metabolism was rewired so that it required lower ATP, decreased CO_2_-emiting reactions, and improved redox balance, using 75% less oxygen than the wild-type strain. Importantly, the engineered strain showed steady β-farnesene production at industrial scale, in 200,000-L bioreactors, thus becoming a viable, cost-effective approach for large-scale production of acetyl-CoA-derived biomolecules [29]. Adaptive evolution of *S. cerevisiae*, in the presence of the isoprenoid pathway inhibitor terbinafine, has recently generated squalene-hyperproducing strains suiTable for industrial scale [31]. The evolved strains presented more than 100 single nucleotide variations (SNVs) in metabolism-related genes, including a point mutation in the *ERG1* gene that encodes squalene epoxidase, responsible for the conversion of squalene into squalene epoxide [31,82]. The approach resulted in a maximum squalene production of 193 mg/L, higher than that of *S. cerevisiae* BY4741 which is commonly used as a source of bioactive molecules in the industrial setting (Table 2; [7,80]). Still, newly introduced triterpenoid-related modifications must compete with the endogenous ergosterol pathway, as yeast uses most of its products in its endogenous metabolism. For example, yeast uses squalene epoxide as a building block of the fungal plasma membrane [83]. Sterol-reduced strains of *S. cerevisiae* have been generated to divert isoprenoid synthesis towards target products [84]. However, excessive blocking MVA pathway, via genetic manipulation, or directed evolution with terbinafine, is toxic and results in cell death. In this context, *E. coli* appears as an appealing model for the manipulation of the isoprenoid pathway, as it lacks any natural downstream pathways or targets for further terpenoid conversion.

Genetic manipulation of *E. coli* has focused on the introduction of key MEP enzymes (such as squalene synthase, SQS) from other squalene-producing microorganisms, via plasmid insertion [85,86]. Further optimization was achieved by the introduction of the full MVA pathway, with modified SQS and HGMR enzymes, from *S. cerevisiae* into *E. coli*, yielding up to 230 mg/L of squalene [86]. Thus far, strategies to generate *E. coli* strains with hyperproduction of squalene have been limited to experimental studies [3,81]. Limitations, such as the need of antibiotics to select transformants, which increases production costs, have hampered the potential of this model as squalene source. Hence, efficient scale-up strategies are required to leverage *E. coli* use in the industrial setting.

**Table 2:** Squalene concentration in different natural sources.

Source	Squalene (mg/100 g)	References
**Natural oils**		
Amaranth	1040–60,000	[3,87,88]
Olive	80–1245	[89,90,91]
Hazelnut	9.3–39.2	[92]
Peanut	27.4–132.9	[89,93]
Corn	10–33.8	[89,94,95]
Grape seed		[89,96]
Soybean	3–22	[93,94,97]
**Distillates**		
Olive	10,000–30,000	[94]
Sunflower	4300–4500	[98]
Soybean	1800–5500	[94,99,100]
Canola	3000–3500	[98]
	**(mg/g DCW)**	
**Yeast**		
*S. cerevisiae (wt)*	0.04–1.6 ^a^	[101]
*S. cerevisiae* *S. cerevisiae BY4741*	1.38 ^b^ 28.4	[101]
*Torulaspora delbrueckii*	0.24	[32]
*Aspergilus nidulans*	0.3	[78]
*Kluyveromyces lactis*	0.6 mg/10^9^ cells	[102]
*Saccharomyces uvarum*	14.3	[103]
(Chang et al., 2008)	70.32	[42]
**Archea ^a^**		
*Halobacterium cutirubrum*	1	[78]
**Bacteria/Protists ^a^**		
*Rubritalea squalenifaciens*	15	[79]
*Pseudomonas* sp.	0.10–0.76	[78]
*Methylomonas methanolica*	1.16	
*Methylococcus capsulatus*	5.5	
*Schizochytrium mangrovei*	0.16	[104]
*Aurantiochytrium* sp. 18 W-13a	198	[105]
*Aurantiochytrium* sp. Yonez 5–1	317.74	[76]
*Aurantiochytrium* sp. BR-MP4-A1	0.57	[104]

^a^ Squalene production in aerobic conditions; ^b^ Squalene production upon fermentation optimization.

## 4. Conclusions

The global demand for bioactive ingredients has significantly expanded in the last decades. Cosmetic and pharmaceutical industries are major consumers of biomolecules and must constantly strive improve standard and innovative formulations.

Squalene (C_30_H_50_), a triterpene from the isoprenoid family of hydrocarbons, was originally isolated from shark liver oil and, since then, its bioactive properties have been immensely explored. Squalene is a biocompatible oil with anti-oxidant, anti-inflammatory, and immunomodulatory properties. Squalane (C_30_H_62_), its fully saturated version, has moisturizing, antioxidant, and UV-protective properties. Due to the lack of double bonds, squalane has more enhanced stability than squalene, and is used in cosmetics as an ingredient to increase the delivery of additional bioactive molecules and products’ shelf-life. Squalene-based oil-in-water formulations are used as vaccine adjuvants that boost immune responses to antigens. To date, three adjuvants containing squalene droplets stabilized by ionic surfactants, MF59, AS03, and AF03, have been included in licenced influenza vaccines, and are now under research for future anti-SARS-CoV2 vaccines. All three adjuvants show immunomodulatory properties and activate both innate and adaptive immunity.

Shark overfishing for squalene extraction has significantly impacted marine ecosystems, leading to the imposition of restrictive environemental regulations to avoid their massive devastation. VegeTable squalene sources appeared as more sustainable alternatives. Nowadays, distillates of olive oil represent up to 40% of global squalene feedstock. However, the need for laborious oil refining processing, extensive culture areas, and geographical variation in weather and soil conditions significantly limit the use of phytosqualene.

Biotechnology advances in recent decades provide a wide range of tools to manipulate the growth and metabolic output of several microorganisms, as well as the ability to maintain large populations in inductrial bioreactors. *S. cerevisiae* and *E. coli* have been used as model organisms in research and industrial settings as sources of bioactive ingredients. Nowadays, *S. cerevisiae* is used to produce squalene at industrial levels. Genetic manipulation of key enzymes of the isoprenoid synthesis pathway, together with optimization of the fermentation process, have been key to generating several hyperproductive strains. The potential of *E. coli* as a squalene source has been recently demonstrated. Although this bacteria strain does not exhibit an endogenous isoprenoid pathway, key related enzymes have been successfully introduced into its genome, resulting in efficient squalene production. Still, it remains to be clarified how industries can benefit from *E. coli* as a putative commercial source of squalene, adding to the already established *S. cerevisiae*. With current and future increasing squalene demands from pharma and cosmetic industries, broadening the range of sources will be key to produce squalene in a competitive yet sustainable and eco-friendly manner.

## Data Availability

Not Applicable.
